# Clinical characteristics and MRI-based phenotypes of perianal abscess formation in children with fistulizing Crohn's Disease

**DOI:** 10.3389/fped.2022.1045583

**Published:** 2022-11-18

**Authors:** Azadeh Tabari, Jess L. Kaplan, Susanna Y. Huh, Christopher J. Moran, Michael S. Gee

**Affiliations:** ^1^ Division of Pediatric Imaging, Department of Radiology, MassGeneral Hospital, Boston, MA, USA; ^2^ Harvard Medical School, Boston, MA, USA; ^3^ Division of Pediatric Gastroenterology, MassGeneral Hospital for Children, Boston, MA, USA; ^4^ Division of Gastroenterology, Hepatology, and Nutrition, Boston Children's Hospital, Boston, MA, USA; ^5^ Takeda Pharmaceuticals International Co, Cambridge, MA, USA

**Keywords:** pediatrcis, Crohn’s disease, fistula, absces, inflammatory bowel diseas

## Abstract

**Purpose:**

The aim of this study was to explore potential correlation of the MR imaging features and clinical characteristics with formation of perianal abscess in children with Crohn's perianal fistulas (CPF).

**Methods:**

From 2010 to 2020, pediatric patients with CPF diagnosis on their first pelvic MRI were identified retrospectively. All patients were divided into two groups based on the presence or absence of perianal abscess. Baseline clinical and MRI characteristics were recorded for each patient. All the statistical calculations were performed using R (version 3.6.3).

**Results:**

A total of 60 patients [F:M 17:43, median age 14 years (IQR 10-15), ranging 3–18 years] were included in this study. Forty-four abscesses were identified in 36/60 children (mean volume 3 ± 8.6 ml, median 0.3 ml). In 24/60 patients with perianal disease, no abscess was detected on the MRI. Ten patients (28%) showed perianal abscess on pelvic MRI at the initial diagnosis. The rate of active disease on colonoscopy (visible ulcerations/aphthous ulcers) was similar in both groups (95% vs. 94%). With regards to disease location, the majority of patients (40/60, 66.6%) in both groups had ileocolonic CD. All patients without abscess had a single perianal fistula (*n* = 24; 3 simple and 21 complex fistulae), however, patients with perianal abscess tended to have >1 fistulous tracts (*n* = 50 fistulas; all complex, 27 single, 10 double and 1 triple). Intersphincteric fistula was the most common fistula type in both groups (79% and 66%, *p *= 0.1). The total length of fistula (3.8 ± 1.7 vs. 2.8 ± 0.8 cm, *p *= 0.006) and presence of multiple external openings (*n* = 25 vs. 7, *p *= 0.019) were significantly higher in patients with abscesses, and fistula length >3.3 cm showed 80% specificity and 83% PPV for the presence of perianal abscess. Fistulas were symptomatic (pain, bleeding or drainage) at similar rates in both groups (68% and 70%, *p *= 0.1).

**Conclusion:**

Pediatric patients with CPF who develop perianal abscess have a distinct imaging phenotype defined by longer fistula length (>3.3 cm), multiple skin openings and multiple fistulous tracts (≥2) on MRI. Patients who have these features but does not have an abscess on imaging may merit more aggressive treatment (and close monitoring) to prevent the development of an abscess.

## Introduction

Approximately 15% of children with Crohn's disease (CD) have perianal symptoms at the time of diagnosis, and 49%–62% develop perianal complications at some point during the course of the disease (1, 2). Despite optimized treatment, almost one-third of patients develop Crohn's perianal fistulae (CPF) and complications such as strictures and abscesses, necessitating surgery within 5 years of diagnosis (3, 4). The presence of penetrating perianal disease complications has been proven to predispose to a greater inflammatory burden. Therefore, significantly impact the quality of life in these children including higher risk of associated psychological distress and disability (5, 6).

Imaging evaluation of the perianal region and accurate depiction of CPF are important for planning treatment strategies. Perianal abscesses often require surgical incision and drainage, potentially followed by seton or catheter placement and antibiotics. The decision to perform surgery, especially for pediatric patients with complex CPF, is based on a number of psychosocial, developmental, and anatomic considerations (7–10). Pelvic MRI is able to identify inflammation and clinically silent abscesses with reported accuracy of 91%, and may enable identification of small abscesses that can respond to antibiotic treatment without surgical intervention (11, 12). In addition, abscess formation in the supralevator space may not be painful or palpable during physical examination (13, 14). For all these reasons, it is important to identify clinical and imaging phenotypes associated with abscess formation. We hypothesized that pediatric patients with perianal CD and fistulizing disease who develop perianal abscess may have distinct imaging-based and clinical features. In this study we aimed to identify MRI features and patient clinical characteristics associated with perianal abscess formation in children with CPF.

## Materials and methods

### Patients

This is a HIPAA compliant, institutional review board approved, retrospective, single institution study. From 2010 to 2020, all pediatric patients, who had perianal CD diagnosis and showed fistulizing perianal involvement on their first pelvic MRI performed for perianal assessment, were identified using an institutional radiology report database. Data were extracted from the electronic medical records, and MR images were then reviewed. Patients were excluded if (1) they did not have a diagnosis of CD based on the colonoscopy, or (2) perianal fistula was not detected on pelvic MRI.

### MRI protocol

MRI examinations were performed using a 1.5 T magnet (HD Excite; GE Healthcare, USA) or on a 3 T scanner (Magnetom Trio; Siemens Healthineers, Erlangen, Germany) with a 16-channel phased-array coil. The standard MR protocol involves a tri-plane localizer followed by triplane fast spin-echo T2-weighted images using a full field of view (FOV) through the entire pelvis, small FOV short tau inversion recovery (STIR) images through the perianal region in the axial and coronal planes, T1-W fat suppressed 3-D gradient echo images before and after administration of intravenous contrast agent in the coronal and axial planes, as well as T1-W fat-suppressed high-resolution 2-D spoiled gradient echo axial delayed images post-contrast administration (15).

### Definition of MRI based features and clinical variables

All pelvic MRI exams were reviewed by a radiologist, blinded to the image findings and disease diagnosis, with subspecialty training in pediatric and abdominal radiology with 12 years of experience reading pelvis MRI. The radiologic features such as: perianal fistula type (based on Parks classification), fistula length (defined as the length from anal canal to skin or the longest measurable distance if the tract does not reach the skin), fistula length above the anal verge (described as distance between anal canal site of fistula egress and anal verge) were recorded. Additional evaluation included the presence of fluid (determined as T2 hyperintense fluid that focally widens the fistula), skin openings and multiple fistulous tracts, perianal abscess (a circumscribed fluid collection with peripheral enhancement >3 mm in diameter in at least 2 dimensions). Abscess volume was calculated using formula: 0.52 × long axis diameter × (short axis diameter) (2). Fistulae were defined as complex if (1) high intersphincteric (IS, Parks' type 1) or transsphincteric (TS, Parks' type 2) fistulae or (2) any suprasphincteric (SS, Parks' type 3) nor extrasphincteric (ES, Parks' type 4) fistulae or (3) presence of abscesses and fistula terminating into the adjacent organs.

Two pediatric gastroenterologists (with 9 and 10 years of experience) blinded to the pathology, independently reviewed the demographic, clinical and endoscopic data from the time of presentation to our institution. Data collected including disease activity (visible ulcerations/aphthous ulcers), CD location and symptomatic fistula (if the patient reported drainage or pain), within 30 days from MRI. Luminal disease was classified according to the Paris classification (16). Diagnosis of perianal disease was established according to clinical symptoms and physical and radiological examinations.

### Statistical analysis

Continuous variables were represented as mean ± standard deviation or median and range and categorical variables as the percentage of the total number. The Fisher's exact test was performed to compare the differences between the categorical variables. Continuous variables were compared using Student's *t*-test. For all of the statistical analyses, a *p* value of <0.05 was considered statistically significant. The optimum threshold for total fistula length discriminating presence or absence of abscess on MRI was calculated based on receiver operating curve analysis (12). All statistical analyses were carried out using R statistical computing software (version 3.6.3, The R Foundation for Statistical Computing).

## Results

### Presentation

A total of 60 patients were included in the study (F:M 17:43, median age 14 years (IQR 10-15). Patients were divided into 2 groups based on presence (*n* = 36) or absence of perianal abscess (*n* = 24). Patients’ demographics and clinical characteristics are summarized in Table 1.

**Table 1:** Demographics and clinical characteristics of the study population.

	Presence of abscess (*n* = 36)	Absence of abscess (*n* = 24)	*p*-value
Mean Age (years)			
Average	11.2 ± 4	13 ± 4	0.15
Median (IQR)	14 (11–15)	14 (12–16)	
Range	6–18	3–18	
Sex (*n*, %)			
F: M	7 (20%):29 (80%)	10 (41.6%):14 (58.4%)	0.08
Mean duration of CD^a^ (months)	2.3 ± 3	2.3 ± 2.7	0.66
Location at diagnosis^b^ (*n*, %)			
L1	4 (11%)	5 (20%)	0.62
L2	7 (19%)	4 (16%)	
L3	25 (70%)	15 (64%)	
Asymptomatic fistula (*n*, %)	11 (30%)	7 (30%)	0.13
Active luminal disease (%)	95%	94%	0.99

^a^ The duration between CD diagnosis and the baseline MRI.

^b^ The Paris Classification: L1, distal ileal + ileocecal location; L2, colonic; L3, ileocolonic; L4, isolated upper disease.

A total of 74 fistulae and 44 abscesses were detected through MRI exams. Seventy percent of fistulae overall were intersphincteric (52/74,) and majority of the fistulae were complex (71/74). With regard to disease location, the majority of patients (40/60) had ileocolonic involvement at the time of CPF diagnosis in both groups.

In 36 patients with perianal abscess seen on MRI, 10 showed abscess at the time of initial CD diagnosis (CD-0). A total of fifty perianal fistula tracts (27 single, 10 double and 1 triple) corresponded to 44 abscesses (average volume 3 ± 8.6 ml) on pelvic MRI. Out of 50 fistulae; 33 were IS, 15 TS and 2 were SS. All fistulae in the patients with abscesses were complex. Twenty-one patients with 26 fistulae had fluid accumulation on T2 images. Three fistulae terminated into the other organs (*n* = 2 into the scrotum, *n* = 1 into the vagina).

In 24 patients, perianal abscess was not observed on MRI. A total of 24 single fistulous tracts (3 simple and 21 complex fistulae) were detected. Out of 24 fistulae; 19 were IS and 5 TS fistulae. Sixteen patients with 16 fistulae had fluid accumulation detected on T2 images. None of the fistulae were communicating with other organs or had external openings. Table 2 shows MRI features of the final cohort stratified by absence/presence of perianal abscess.

**Table 2:** MRI characteristics of the study population.

Variables^a^	Presence of abscess	Absence of abscess	*p*-value
Subtypes of perianal fistulas (*n*, %)			
Intersphincteric	33 (66%)	19 (79%)	
Transsphincteric	15 (30%)	5 (21%)	
Suprasphincteric	2 (4%)	–	
Number of fistula tracks (*n*, %)			
1	27 (54%)	24 (100%)	**0.027**
2	10 (40%)	–	
3	1 (6%)	–	
Number of fluid accumulations (*n*, %)			
Patients	21 (58%)	16 (66%)	0.5
Complex fistula (*n*, %)	50 (100%)	21 (87.5%)	
Communicating with other organs (*n*)	3	0	
Multiple skin openings (*n*, %)			
Patients	22 (61%)	7 (30%)	**0.019**
Fistula	25 (50%)	7 (30%)	
Average fistula length above the anal verge (cm)			
Average	1.8 ± 1	1.65 ± 0.7	0.09
Median	1.7	1.7	
Range	0–4.7	0–3.1	
Average total fistula length (cm)			
Average	3.8 ± 1.7	2.82 ± 0.8	**0.006**
Median	3.3	2.75	
Range	1.1–7.6	1.2–4.3	

^a^ At the time of baseline MRI positive for perianal fistulizing CD.

Bold values indicates *P* < 0.05 and was considered as significant.

### Overall outcome

Among the MRI features examined, total perianal fistula length (3.8 ± 1.7 vs. 2.8 ± 0.8 cm, *p *= 0.006) and number of external openings (*n* = 25 vs. 7, *p *= 0.019) were found to be significantly higher in patients with abscesses. Total fistula length <3.3 cm was shown to have a significant correlation with lower risk of abscess formation (sensitivity = 50%, specificity = 80%, positive predictive value (PPV) = 83%, negative predictive value (NPV) = 43%).

## Discussion

The present study is the first study to combine clinical and MR imaging features associated with perianal abscess formation in pediatric patients with CPF. For the management of CPF it is essential to locate the fistula origin, establish the anatomical course of the fistula tract and exclude or identify the presence of associated perianal abscess (17–21). Patients with CPF who develop perianal abscess may subsequently require major operative intervention (1, 22). Studies to determine the appropriate timing of operative intervention for children with perianal abscess are lacking, leading to significant variability of treatment plans based upon surgeon preference and experience (23–25). Thus, early rule out of presence of perianal abscess is essential and should be discussed with the surgeon when drainage is detected.

In our analysis on perianal abscess in children with CPF, intersphincteric fistulae were the most common (66% on MRI) and transsphincteric fistulae were the second most common (30%) types. There were no extrasphincteric fistulae in the present study. In previous studies on adult population with CPF, transsphincteric fistulae were most frequently found (25, 26). In our study, one possible explanation for the finding that intersphincteric fistula was the most common type might be that for pediatric patients, the duration of disease was relatively short as compared with that in adults. Patients who develop perianal abscess were more likely to show perianal fistula with ≥2 fistulous tracts (*n* = 31, *p *= 0.027), and multiple skin openings (*n* = 22*, p *= 0.019). In addition, the fistula length was significantly shorter in patients who did not form perianal abscess (*p *= 0.006). A study by Choshen et al. highlighted that the best imaging findings indicating the severity of pediatric perianal CD were presence of collections >3 mm, location and length of the fistulas (27). Also, a previous study on MRI predictors of treatment response in children with CPF reported that maximum fistula length ≥2.5 cm was a predictor of disease progression (15). Our study extends that work by identifying features associated with abscess formation. A fistula length cut-off of <3.3 cm for perianal fistula showed 80% specificity and 83% PPV in patients with perianal abscess.

In this study, 16/60 (26.6%) of patients with perianal fistulae and 10/36 (28%) of patients with perianal abscess were CD-0 (presence of abscess at the time of initial CD diagnosis). Although not significant because of the small sample size, the patients who developed perianal abscess were more likely to be males (80% vs. 58.4%) and younger. This may be in part due to the known greater male > female predominance of pediatric Crohn's disease in the pre-pubescent age group. A study by Short et al. demonstrated that children who presented with perianal perforating CD at the time of diagnosis, were mostly young (mean 9.3 years old) and males (81%) (28).

The prevalence of perianal and anorectal abscesses, in general, are underestimated, since most patients do not seek medical attention, or are dismissed as having asymptomatic CPF. In situations where the child has perianal pain and discomfort and the abscess formation is suspected, prompt evaluation under anesthesia, including drainage is the procedure of choice to minimize damage to the sphincter. However, we found no correlation between the patients having symptomatic fistula and the presence of a perianal abscess (*p *= 0.1). Moreover, there was no significant association between the activity of luminal CD and development of perianal abscess in our patient population.

Strengths of our study include describing both MRI and clinical characteristics of patients with CPF associated with perianal abscess formation based on one of the largest pediatric datasets. The limitations were single-center retrospective nature of the study. In addition, a few patients were excluded because imaging was performed after specific surgical procedure/medical therapy to decrease the heterogeneity of the data. Additionally, our analysis focused on individual MRI features of CPF (e.g., fistula length, location, complexity) rather than scoring systems of perianal fistula severity that have previously been reported. Finally, a single radiologist (our institution's local expert on perianal fistula MRI interpretation) reviewed all the MRIs, and further studies on patients undergoing serial MR imaging exams reviewed by 2 independent radiologists would be helpful to assess the predictive value of these imaging features in terms of clinical management.

## Conclusion

MRI quantitative parameters including fistula number and length have a high association with abscess development and should be reported when interpreting these imaging exams in pediatric patients with Crohn's disease. Given these findings, patients who have these features but does not have an abscess on imaging may merit more aggressive treatment (and close monitoring) in order to prevent the development of an abscess.

## Data Availability

The original contributions presented in the study are included in the article/Supplementary Material, further inquiries can be directed to the corresponding author/s.
